# Reproductive health care appointments: How the institutional organization of obstetric/gynecological work shapes the experiences of women with female genital cutting in Toronto, Canada

**DOI:** 10.1371/journal.pone.0279867

**Published:** 2023-01-19

**Authors:** Danielle Jacobson, Daniel Grace, Janice Boddy, Gillian Einstein

**Affiliations:** 1 Dalla Lana School of Public Health, University of Toronto, Toronto, Canada; 2 Department of Anthropology, University of Toronto, Toronto, Canada; 3 Department of Psychology, University of Toronto, Toronto, Canada; 4 Department of Gender Studies, Linköping University, Linköping, Sweden; Universidad Veracruzana, MEXICO

## Abstract

We investigated the social relations shaping the reproductive health care experiences of women with female genital cutting (FGC) in Toronto, Canada. Using Institutional Ethnography, we interviewed eight women with FGC and seven obstetrician/gynecologists (OB/GYN). We found a disjuncture between women’s needs during appointments that extended beyond the reproductive body and range of care that doctors were able to provide. Women engaged in emotional healthwork during appointments by explaining FGC to doctors, reading doctors’ body language, and getting through vulvar/vaginal examinations. Women reported that if they had emotional reactions during appointments, they were often referred to a mental health specialist, a referral on which they did not act. OB/GYNs described their specialty as “surgical”—training centered around treating reproductive abnormalities and not mental health issues. Therefore, the disjuncture between women’s needs and OB/GYNs’ institutional training highlights the difficulties inherent when bodies of “difference” encounter the reproductive health care system.

## Introduction

Female genital cutting (FGC) involves the cutting of the external genitalia. It is practiced across Africa, the Middle East [1], and Asia [2]. There are multiple types of FGC, ranging from type I to IV, with type IIIb (cutting and stitching together of the labia majora) considered the most severe; although a higher number, type IV is not the most severe as it broadly entails all other non-medical, harmful female-genital procedures such as “pricking”, “piercing”, and “incising” [1, p.1].

The World Health Organization estimates that there are over 200 million women with FGC worldwide [3], many of whom have immigrated and settled in host countries across the west [4]. Although there have been no official estimates of prevalence in Canada, it has been reported that over 20 000 people from practicing countries (where at least 90 percent of women and girls have FGC) immigrated to Canada from 2005–2009 [5,6].

As women with FGC build their lives in host countries, they encounter difficulties when seeking out health care. In Canada, women with FGC have reported receiving upsetting remarks from doctors and feeling stigmatized and shamed during care [7,8]. However, little work has been done on health care system interactions for women with FGC in the Toronto, Ontario context since the early 2000s [7,8]. More generally, western health care experiences for women with FGC have included: fear of caesarean section [9–11]; not disclosing health issues to providers out of shame and fear of judgement [12]; and hesitancy to seek health care in a timely manner (especially when in labour) [13]. These health care behaviours and experiences are important because if a woman with FGC-related conditions (i.e., chronic UTIs, keloids, abscess, clitoral neuroma, bacterial vaginosis) [14] hesitates to seek reproductive health care, an opportunity for early care may be missed.

Even though it is known that women with FGC face multiple difficult reproductive health care experiences, these challenges have not been considered in relation to doctors’ professional training. Across western countries, doctors may not be adequately trained on the particular health needs of women with FGC [15–20]. Even though 71.5% of Italian health care professionals treated women with FGC, more than half had no specialized training [18]. Forty percent of Spanish physicians were unable to distinguish between FGC types [16]. In Belgium, only 11% of doctors reported having been educated about FGC [15]. In the United States, almost half of doctors studied lacked training on how to treat women with FGC [20]. Given this wide lack of training across the west, affected communities often view doctors as unfamiliar with caring for their loved ones with FGC [21].

While some women with FGC who participated in this study referred to these practices as “female genital mutilation” (FGM), some referred to them as “circumcision” and others as “cutting” (FGC). Women we spoke with often used multiple terms to describe the practices within one interview; however, we refer to the practices as “cutting” to denote physical cutting of the genital region. The naming of FGC is complex and it is our priority to maintain respect and inclusivity for the many perspectives of women who have FGC with the use of this term.

In this study, we explored the social relations that shape the reproductive health care experiences of women with FGC in the Greater Toronto Area, Canada (GTA). “Reproductive health care appointment” refers to any health care appointment that involves care of a woman’s reproductive system carried out by an obstetrician/gynecologist (OB/GYN). Social relations go beyond relationships between people and include connections among people’s activities within an institution [22,23]. The social relation explored in this study is the institutional organization of obstetric/gynecological work, including OB/GYNs’ knowledge and expectations, professional standards, and organizational rules [24]. It is within this context that women with FGC obtain reproductive health care.

This study arose out of meetings for women with FGC at a local community center in the GTA. Here, women voiced concern about their doctors’ professional training to care for them and expressed how they were treated poorly during reproductive health care encounters. This motivated us to study how their experiences were shaped. These meetings sparked questions surrounding the reproductive body with FGC as a boundary object that “resides between social worlds”—one of women with FGC and another of the health care professionals who treat them [25,26 p.604]. From an Institutional Ethnographic perspective—the sociological approach used in this study—women with FGC traverse these different social worlds, each with varied ideologies, discourses, and normative practices. In the everyday social worlds of women, FGC (at the time of the original cutting in natal countries) allowed them to *conform* to societal body norms. However, upon immigration in diaspora, the very same practice was illegal and a *source of stigmatization and difference* [4,27–29].

## Methods

This research is part of a larger study investigating the social relations that shape women with FGC’s reproductive health care experiences in the GTA. The GTA includes the city of Toronto and four regional municipalities (Durham, Halton, Peel, and York). Further, the health care system in Canada is publicly funded and universal. In Ontario, the Ontario Health Insurance Plan (OHIP) covers most basic health care appointments required for medical reasons.

In reporting the current study, we have consulted the Consolidated Criteria for Reporting Qualitative Research (COREQ), with most checklist items considered [30]. This study received approval from the University of Toronto Research Ethics Board.

### Institutional ethnography

Institutional Ethnography (IE) was created by Dorothy E. Smith [23,31,32] and is unique in its pursuit to study how social institutions (e.g., the education system, the medical institution) shape people’s (e.g., students, patients) experiences [31]. To understand how people’s experiences happen within any particular social institution, IE looks beyond the individual or any individual experience to the societal level. This means that researchers do not look to the people themselves as the source of their everyday difficulty but rather to social institutions and the way that they operate [33,34]. The goal is to understand what happens in people’s everyday lives and then to investigate how those experiences are fostered by, for example, the guidelines, policies, laws, and training of professionals within those institutions. It is this shift of focus from the individual to the larger social level that differentiates IE as a critical research strategy from other research approaches that investigate people’s perspectives on certain issues [31].

We used IE to investigate the institutional and social relations that shape women with FGC’s reproductive health care experiences, including their interpersonal interactions with care providers. Our goal upon entry into the research was to understand how women with FGC’s everyday health care concerns and experiences were shaped by the Canadian medical institution.

#### Extending the notion of healthwork

Central to this study is the notion of “healthwork”—an extension of Smith’s [31] generous notion of work. “Work” draws attention to the everyday actions of people and orients all actions as “a part of actual local practices” [35 p.159]. This notion was extended by health studies scholars to direct attention to the particular work involved in looking after one’s health (i.e., “healthwork”) [36–38]. In a previous publication from the larger study, women with FGC engaged in healthwork that centered around emotion to prepare for health care encounters. We described this as “emotional healthwork” [39], extending the notion of healthwork and directing attention to the emotional work burdens navigated by women with FGC that allow them to “look after their health” [36 p.18]. While any person inhabiting a body outside the white, male, unscarred prototype could find teaching a doctor about their body exhausting and emotional, women with FGC present a unique case in that they are black, immigrants, and carry on their bodies a gendered practice that is often stigmatized in the western world [39].

Explicating the emotional healthwork that women with FGC engage in to navigate reproductive health care is important as it allows for an understanding of not just what women experience, but what material actions they take during the health care encounter. After engaging in work to prepare for appointments [39], participants’ emotional healthwork does not cease; it instead carries forward into the clinical interaction.

Emotional healthwork differs from concepts such as “emotional labour”. As Hochschild [40] described, with emotional labour, health care personnel conceal emotions elicited by the patients’ difficulties as an aspect of job performance. While engaging in emotional healthwork, one’s goal is not to appear a particular way for someone else, but rather to look after one’s own health by engaging in work to attend or complete the health care encounter.

We do *not* characterize women as inherently or exceptionally “emotional”. We view emotions as a “medium through which people act and interact” [41 p.399]. Emotions elicited in the care context therefore provide another means to understand the extra and inequitable work in which women with FGC often must engage. Emotional healthwork is not a reflective trait of any particular people and could apply to anyone navigating the health care system with any body not considered “normal” [39].

### Research participants and recruitment

A community advisory group (CAG) including two women with type IIIb FGC in the Toronto community was formed at the outset of the research to provide consultation on the project. The CAG met with the first author multiple times throughout the research process and helped to advise the direction of the project. The CAG provided cultural insight into the interview approach, gave direction on how the project could be useful for the community, and also provided consultation and feedback on the findings of this study. While the CAG did not represent the entirety of the varied views and perspectives of the vibrant, diverse community of women with FGC in the GTA, discussing the findings with the CAG helped to ensure that the results resonated across the CAG’s unique life experiences.

Inclusion criteria for women with FGC included: having type IIIb FGC (determined via self-report), being over 18 years of age, and having accessed reproductive health care in Toronto or the GTA no more than 10 years prior to the interview. Doctors were included in the study if they were a certified OB/GYN, had practiced medicine in Toronto or the GTA, and had prior medical experience with women with FGC. Doctors met women with FGC in the context of medical appointments arranged by women with FGC.

To avoid a conflict of interest for women with FGC and doctors, doctor-patient dyads (women’s doctors and doctors’ patients) were not included. Women with FGC and doctors were unknown to each other. Participants in this research were situated in a societal context that may have influenced their perspectives on FGC. For example, in Toronto and the GTA, Canada, FGC is stigmatized by a broad societal discourse that opposes FGC (i.e., anti-FGM discourse), resulting in the stigmatization of the practices, women themselves, and related practicing cultures [39]. In this same context, laws against FGC, which are partial to the cosmetic genital surgeries elected to by Western-born adults, have been found to foster ethical dilemmas for OB/GYNs caring for women with FGC [42]. Further details about the larger study have been reported [39].

Recruitment was carried out by referrals from a community health centre serving the GTA. This was an opportunistic cohort. Recruitment was an open-ended process. Participants either reached out via email or telephone to the first author after seeing a flyer or being referred, or the first author reached out to participants who agreed to have their information shared by the community health centre.

Eight women with FGC (from Sudan, the Gambia, Somalia, and Kenya), aged 25–70, who immigrated to Canada between 1993 and 2019 and seven obstetrician/gynecologists (OB/GYNs) participated. Five of the seven doctors were born in Canada, were between the ages of 35–75, and received some part of their medical education in Canada. The doctors had practiced medicine for between six and 40 years. Six were women and one was a man.

### Interviews

The first author, a cis-gendered, Canadian-born woman, conducted all the interviews. For all interviews, we used a semi-structured interview guide—one for women with FGC and another for doctors—iteratively developed in consultation with the CAG. Interviews began by asking participants about what was and was not going well during reproductive health care appointments. Open-ended questions about their experiences prompted them to detail step-by-step what took place during appointments, which revealed their concerns. They sought care for pregnancy and childbirth, routine tests (i.e., vulvar/vaginal examinations), and help with reproductive problems (i.e., pain).

We then interviewed doctors and asked them about their experiences caring for women with FGC. Open-ended questions about their experiences prompted doctors to describe what happens when a woman with FGC walks into their office seeking care. Some had only treated a few women with FGC, while others had treated many.

Each interview was between one to two hours long. All interviews were audio-recorded, transcribed, and all transcriptions were verified by a dual reading by the transcriber and the first author. During interviews, we engaged in the IE technique of checking and re-checking our understanding of participants’ accounts. We did this by repeating what participants said, asking if it was correct, and either solidifying or readjusting our interpretation based on their feedback [24]. We also followed up with some participants after conducting iterative analysis to check our understanding as it developed across participants’ accounts.

For participants’ comfort and convenience, interviews were held where requested (i.e., at a community health-centre, hospitals, University of Toronto, and by phone). All participants completed written informed consent and sociodemographic forms prior to participating in the study. Two who were more comfortable with a language other than English were provided an interpreter. The interpreter was trained on anonymity, confidentiality, and the study’s purpose, and then interpreted the interview in real time.

### Analysis

The first author led the analysis. The approach to analysis was iterative with collected data directing the focus of subsequent data collection. This means that interview questions evolved and developed as we learned more from participants’ accounts about their experiences.

Through the data analysis we traced the social organization of women’s reproductive health care experiences. We used transcripts as texts, iteratively reading, re-reading, interpreting, and writing our thoughts through this process [43]. Indexing was also utilized. This involved grouping together particular types of work described by participants [35]. For example, some women with FGC described the work of explaining their FGC during health care appointments. References to this type of work across participants were then grouped together. These analytic processes helped us better understand the connections between people and the institution [44,45].

While the CAG was not involved in the primary analysis of audio recordings and transcripts, results derived from the analysis were brought to the CAG for discussion and reflection. Both women with FGC and doctors were invited to separate focus groups to discuss the developing results of the study and the ways in which the results resonated with their experiences. Perhaps due to hardships presented by the COVID-19 pandemic, only one woman with FGC and two doctors were able to attend the respective focus groups.

## Results

### Emotional healthwork to navigate reproductive health care

At the reproductive health care appointments, emotions were provoked when participants had to explain their FGC, when reading doctors’ body language in response to their own body, and from vulvar/vaginal examinations.

#### Work of explaining FGC to health care personnel

Participants were warned about what might happen during appointments from family and friends’ unsatisfactory experiences [39]. Knowing that FGC was stigmatized in the popular imaginary [39], women would explain their FGC at the appointment’s outset to prepare doctors to treat them:

I explain before [the doctor views my vulva], I do not surprise them [the doctor]…I have to do a lecture for them…I say, this is what happened, and this is why you need to be careful with me… [Often doctors] don’t know. So, I become the teacher…You have to explain yourself over and over and over again.

(Asha)

Asha believed that doctors were not well-trained to treat bodies with FGC and were “surprised” when viewing her vulva. Providing crucial information to doctors to improve the medical encounter demonstrates women’s expertise and savvy in navigating the system. This work of strategizing how to get the most out of the health care encounter and of teaching their doctors was described as “exhausting” and “emotional”.

While some participants with FGC strategized their teaching to make appointments go smoother, others were actually required to explain because their doctors, unfamiliar with the practices, requested more information. Tim said, “[The OB/GYN] asked me to explain more, because she doesn’t know much about it…And I was just filled with emotions.” Other times, women with FGC were asked by doctors to explain their bodies as a part of a clinic’s intake protocol. Often, participants with FGC were required to explain the practice to multiple health care personnel.

Tim explained how each health care worker she encountered was unfamiliar with FGC and wanted to hear her history directly from her, rather than relying on the intake notes documented by the previous personnel: “They want to hear it from the horse’s mouth. So, I have to explain my entire story from beginning to end…it is very emotionally exhausting, because it’s like, you are repeating yourself over and over again.” In this case, she had to describe her FGC history to an intake nurse, then to a general practitioner, then, after being referred to an OB/GYN, had to explain again to that intake nurse and once more to the OB/GYN. Often, women in this study had to teach four different people about FGC before being given the care they sought. Women with FGC said that this work of explaining was “usually the emotional part”, underscoring that emotions were elicited by the clinical encounter.

#### Work of navigating doctors’ body language

Participants with FGC also described picking up on subtle social cues like doctors’ body language, which made them feel stigmatized. Lailatou said, “It is the body language, the gesticulations…this is one of the major problems. Their talkings are nice, but then you find what they say definitely doesn’t match with the body language.” Although Lailatou’s doctor’s words were assuring, her discomfort stemmed from this verbal communication not being congruent with her doctor’s bodily communication, which she viewed as a “major problem.”

In particular, women with FGC described a “look of shock” on their doctors’ faces when viewing their vulvas. For example, Tim said, “I wasn’t feeling that much comfortable…I could tell she was a little bit with the surprised face”. Lena described becoming aware that doctors’ implicit reactions indicated that her body was “different”: “In our [natal] country, because all the females go through this, it is normal. But, when I came here, I felt that I am different. Because of the reactions of the people, like the doctors’ reaction.” Women had thus felt “normal” until they experienced the bodily reactions and facial expressions of western doctors upon learning about or viewing their FGC. Hodan further explained:

We are looking for acceptance. Don’t look there and judge me. I am a human being…When the body language of the doctor is not a good one, we feel sad, sometimes we ask, ‘why is he doing that?’…The facial expression also plays a role in how I feel…It is an issue we always go through.

Women described how doctors communicated a judgement based on their FGC without words, which in turn led to participants managing emotions such as sadness during their reproductive health care appointments.

Women in this study accepted their own bodies and expected the same from the reproductive health care system. Lailatou explained, “It’s an integral part of me now. I cannot erase it…It’s just part of me.”

#### Work of getting through vulvar/vaginal examinations

Women also described how unpleasant memories came up during vulvar/vaginal examinations. Lailatou said: “It is always emotional when I have this physical connection…It brings back all these crazy memories.” It evoked memories of the original cutting, given the similar position of women’s bodies during the original FGC and the examination. Both involved lying on one’s back, with genitals exposed. Tim said, “I wasn’t comfortable…There are some memories that come back…it’s like you lay down again.” Women communicated that it was the examination itself that elicited these memories and expected that their carers would consider this context. Rama explained that doctors “need to be aware [and] need to know what to do” when faced with women with FGC.

Participants recounted that when they had emotional reactions, their doctors did not know how to deal with them. In turn, this resulted in referral for mental health care. Lailatou said, “I think in her mind because I was crying and everything, it was emotional like it always is. So, she was like, ‘Oh, yeah, you might definitely need perhaps some kind of counselling.’ And I am like [gasps].” The gasp signifies being overwhelmed by the referral to counselling for a response that Lailatou viewed as a result of the care at hand. Though women’s responses may have also been related to previous trauma on account of their FGC, women emphasized that it was particularly their needing to explain their FGC, their doctors’ body language, and referral to mental health care that elicited these emotions.

#### Hesitancy to follow up

Women with FGC who participated in this study completed their scheduled appointments, however, because they felt uncomfortable, they hesitated to follow up and undergo routine tests. Lailatou continued, “I was supposed to go back. And, somehow, that never happened…I was supposed to follow up, and I never do…I just don’t want to be there again.” Some participants said that discomfort from vulvar/vaginal examinations dissuaded them from electing to have another one in the future. However, others described that it was discomfort from doctors’ body language that deterred them from following up more generally:

When I go to see a doctor, I know I am circumcised. And, I tell the doctor that I have FGM. Although I already give the introduction, if I see the doctor’s body language change, I feel very bad, I regret coming in. And, I might not come back and see a doctor until it [ailment] is too bad.

(Hodan)

When women reported not getting what they needed during appointments, they took action by avoiding a system they perceived was not fully prepared to care for them.

### Linking up emotional healthwork with the institutional organization of obstetric/gynecological work

In this section, we link women’s emotional healthwork to the organization of obstetric/gynecological care work, exploring the institutional conditions beyond any single OB/GYN that shape participants’ experiences. The obstetric/gynecological care work that we found to shape women with FGC’s reproductive health care experiences included (1) the scope of obstetric/gynecological care, (2) the notion of returning the body to “what it’s supposed to look like”, (3) the differentiation of types of emotional care, (4) limitations of time, and (5) the surgical focus of the obstetric/gynecological specialty.

#### Differentiating obstetric/gynecological work from other specialties

Part of women with FGC’s emotional healthwork was elicited by the scope of care that OB/GYNs were able to responsibly provide. Dr. Rose explained, “When I see something, I’m like, ‘oh this is good in my scope’, or ‘this is maybe better for somebody else’…I’m pretty confident in what I can and cannot do.” The institutional language of “scope of practice” helped OB/GYNs to determine what fell in and outside the category of knowledge in which they had expertise. This was an important distinction since practicing medicine outside of their scope of practice could lead to being reprimanded professionally.

To define the kind of care for which doctors were accountable, or what was within their scope, patients’ needs were categorized within particular subsets of medicine. These subsets each reflected a different body system. Dr. Gold explained how they were not well suited for other subsets of medicine (like psychiatry, reflecting the mental health system) which they did not professionally pursue: “[Women] should get the complex of care they require. And, personally, I am not a psychiatrist for a reason. I am not a social worker for a reason. I am not a psychologist for a reason.” This differentiation helped participating OB/GYNs to have a strong conceptualization of their particular affinities and skills, which were suited specifically to the medical specialty and body system they pursued—obstetrics/gynecology.

OB/GYNs explained that they were skilled within the obstetric/gynecological category of medicine and had a responsibility not to provide care for body systems that were the purview of a different category of medicine in which they were not trained (e.g., mental health care):

The psycho-social aspects…we don’t get training in that. That’s through nursing and psycho-social and social workers. That is not a component of gynecology…As gynecologists, we’re actually surgeons. And we deliver babies, right? And we deal with menstrual abnormalities and vulvar abscess. So, it’s more the physical aspects. So, we don’t normally deal with the psycho-social.

(Dr. Rose)

OB/GYNs felt it important to refrain from providing care for bodily systems for which they were not trained to treat. Therefore, the limits of the obstetric/gynecological scope of care ultimately shaped the kind of support women could receive and hence their emotional healthwork.

#### Returning the body to “what it’s supposed to look like”

Smith [31] describes how institutional discourses (e.g., those used in the training of OB/GYNs) reduce the complexity of the everyday world, helping doctors to deduce an illness from a complex set of bodily symptoms. OB/GYNs were trained to recognize genital anatomy, appearance, and function that varies from the very wide range of what is considered to be “normal”. Dr. Gold described: “I could look at a vulva and say, ‘This is not correct’. And if somebody wants it fixed, I could figure out how to fix it…I know the anatomy, so I know what it’s supposed to look like.”

Being able to recognize variations from “what it’s supposed to look like” and “fix it” involves simplifying complex systems to recognizable problems with tested solutions. The objective is to reduce human suffering. For example, Dr. Cudjoe described how they were able to recognize labia that were sewn together, determine the source of pain, and make a treatment plan to resolve it:

She had a little pinpoint, literally a pinpoint opening, where the vagina was. The rest of the area was completely obscured by scar tissue…It was obviously why she was having pain because there was considerable scarring in the area. So, we did a procedure to get rid of the scarring and open up the vagina.

To determine the source of the patient’s pain, Dr. Cudjoe had to focus in on the patient’s vulvar anatomy, thereby reducing the complexity of its intertwinement with the rest of the body and the patient’s social world. Dr. Cudjoe felt prepared to do procedures like these based on their training on how to “see a process that is interfering with the usual anatomy, [and] know how to treat that process so that the anatomy is restored”. Thus, obstetric/gynecological training, scope of expertise, and surgical work organized the aim of “restoring” the body to its “usual anatomy.”

#### Differentiating between types of emotional care

It is not that the OB/GYNs interviewed *did not want* to support women’s emotional needs, but that they often felt they *did not have the expertise* to do so. As Dr. Cudjoe put it, “I’m a gynecologist. My expertise is in the surgical treatment of these women. I feel comfortable doing it. Listening to their emotional trauma, their family problems and so on, is beyond my expertise.”

While doctors emphasized their lack of expertise in addressing trauma, they also made a critical distinction between different types of emotional care—both of which we consider as care work and not healthwork (the work that someone does to look after their own health). Dr. Beatrix explained how they were skilled at supporting difficult emotions, “Emotions are kind of part of what we deal with our patients. All day and every day. And, no one will say, ‘Oh, I don’t know how to deal with difficult emotions.’ That is what we do, it’s our job.” This type of emotional care, which was considered as part and parcel of obstetric/gynecological work, included measures of sensitivity like reassuring women by saying they had seen FGC before, paying attention to and allaying fears, and performing comforting gestures:

I cover people when I’m doing an exam…I try not to look at their private parts for very long. I get my fingers where they go and then I look over here, or over here or–you know. I do a bunch of different things to be respectful.

(Dr. Janice)

OB/GYNs therefore viewed part of their role as providing general emotional support.

However, there was an added layer of complexity, given the cultural meanings behind the FGC practices, and some OB/GYNs were concerned with stigmatization and mental health:

It isn’t the emotions, it is the cause of the emotions that makes it something the person [doctor] would reasonably say, ‘I don’t have the depth of knowledge here to understand these issues’…In which case, we would be outside of our scope because now you’re helping someone deal with things that are not your kind of qualification…[When] there’s [a] situation impacting their life in a more profound way, that’s where you would have to refer that person to get more help with dealing with that, because that deals with mental health and stigma.

(Dr. Beatrix)

This second kind of emotional care in which patients’ emotions were perceived as more complex and intertwined with culture and stigmatization was viewed as separate from OB/GYNs’ role in general emotional support. These emotions (related to stigmatization) were viewed as best addressed under a different category of medical training, reflecting a different bodily system (i.e., mental health) that OB/GYNs interviewed did not feel qualified to provide.

OB/GYNs viewed care as complex, requiring a team. Dr. Gold described, “Everything we do in all elements of care is patient specific, situation specific and ideally holistic. That doesn’t mean an individual provider is doing all that. So, you have a team when patients come in for care.” However, from women with FGC’s accounts, there were no mental health care specialists on hand. OB/GYNs provided referrals to mental health specialists who were trained within that particular category of care, but not necessarily on FGC. Dr. Rose said, “We are not psychologists so if something comes up it is not something we would be able to manage…So, we refer to social workers and the people who deal with trauma.” This aligns with women with FGC’s accounts; they received, but did not take up, referrals to mental health care when they had emotional reactions to the doctor’s body language, questions, or examinations.

#### Limitation of time

The organization of obstetric/gynecological work and distinguishing of different types of emotional care was not only determined by the “scope” of obstetric/gynecological care (i.e., surgical training and focus on the reproductive body), but also by time limitations. Dr. Rose explained, “With our jobs we do not have the time [to address emotions] because we are dealing with the physical aspects…I would love to do it, but it is not in our scope.” Dr. Janice described wanting to help with more complex emotional aspects but indicated that the limitation of time prevented their ability to do so: “You just want to do everything for them [patients with FGC]. And you can’t. You’re not the social worker. You’re just the obstetrician who has 30 people sitting in your waiting room, while you’re spending more and more time with social issues.” Since time was a scarce resource, OB/GYNs could commit to only providing care considered to be within their particular obstetric/gynecological scope.

When appointments inevitably went longer than scheduled, doctors made up this time by giving up their breaks:

With all doctors, the limitation is time…My Wednesday was 8 to 6 straight. I had 5 minutes for lunch. I wasn’t able to eat my lunch in 5 minutes…It’s just limitations of time. There are just so many patients…But, visits are half an hour.

(Dr. Rose)

Because OB/GYNs had more patients than there was time for, and limited time per patient, they sacrificed their personal time so that they could address as many patients as was necessary. Being aware of the limitations of the medical system beyond any single doctor, whether regarding the boundaries of their specialty or the navigation of what can be reasonably accomplished in one appointment, contributed to the institutional organization of obstetric/gynecological work. This made it important for OB/GYNs to focus on priorities considered within their expertise.

OB/GYNs were trained to operate within and facilitate the system to ensure that all patients got access to timely care. Dr. Gold described one example of this when a woman with FGC visited multiple OB/GYNs for the same issue:

I saw a woman for her fifth opinion! So, I asked her, ‘What happened with your other visits? How come you needed to see a fifth gynecologist for the same issue?’ She didn’t feel a good fit with one of them, she didn’t feel heard with one of them…It is bad use of the health care system.

This “bad use of the health care system” exemplifies that women with FGC were frustrated from not feeling heard which exacerbated their emotional healthwork. This often led women to do more work to find a different doctor who could help them. Here, the institution was in view when considering the patient’s navigation of care rather than the reasons why they saw five OB/GYNs for the same issue. OB/GYNs therefore had to have the functioning of the institution in view in order to facilitate care for all patients.

## Discussion

To better understand the social relations that organize women with FGC’s reproductive health care, we asked women with FGC in the GTA about what was and was not going well during their reproductive health care appointments. Women with FGC highlighted the emotional healthwork they did to get through appointments. This work included explaining their FGC, which they described as exhausting and emotional. It also included picking up on social cues like doctors’ body language, which often made them feel stigmatized. Women also worked through emotions to get through vulvar/vaginal examinations. Women with FGC had thought deeply and practically about their reproductive health care experiences, communicating this with strength and pragmatism. They were savvy in their navigation of care, recognizing gaps in doctors’ knowledge, strategizing, and engaging in work to get the most out of the appointment.

It is possible that, given the scope of this research study focused on reproductive health care experiences, women who had experienced difficult health care encounters self-selected to participate. Further, one woman with FGC and two doctors attended the respective focus groups after all data was collected, perhaps due to hardships presented by the COVID-19 pandemic, which allowed us to reflect on findings with only a small group of participants.

The emotional healthwork in which women with FGC engaged was shaped by the institutional organization of their reproductive health care, namely the organization of obstetric/gynecological work practices. OB/GYNs interviewed described a surgical emphasis in their training, which organized their scope around the reproductive body. This shaped the emotional healthwork that women engaged in, as emotional responses related to what was viewed as stigmatization and mental health were not within the range of care that OB/GYNs were able to provide.

Although previous literature generally acknowledges the role of emotions in reproductive health and health care [46–49], the current research links related emotional work to the *organization* of care beyond any single provider. While previous ethnographies aimed to study emotions [41,50,51], this was not our original focus. However, the emotions that women had to work through to navigate appointments could not be ignored and led us to look at the structure of the care. Thus, IE allowed us to start with the emotions and follow the thread to how obstetric and gynecological work was institutionally organized. Findings that centre around emotion may not have been uncovered with the use of other approaches to inquiry [41] since ethnographies (in particular, IE) allow for the uncovering of “unseen work” [p.398].

In previous IE studies, emotion has not often been the starting point. When emotions have been brought up in previous ethnographies, they have not been discussed as an aspect of healthwork itself. For example, one study did discuss emotions in terms of the “personal challenges” and “emotional effects” related to prenatal care for individuals living with HIV [52 p.139]. However, this emotional difficulty was not discussed as related to healthwork. Individuals living with HIV may too experience emotion during appointments (including testing and diagnosis), given their stigmatization and exhausting healthwork [36,53–55]. This aligns with our view of emotions and related work as phenomena experienced by any person navigating care with a body that deviates from what is considered biomedically “normal”.

The healthwork that women with FGC in this study described had further consequences. When their emotional work was not addressed during the appointment, women became hesitant to return to doctors or they shopped around, seeking out many doctors. There has been minimal exploration of the health care seeking behaviours of women with FGC in the literature, with the exception of few articles that report women’s hesitancy to obtain timely care when in labour [13,56] out of fear of what may happen to their bodies in a western care context [13,57]. Other studies have also found that not feeling heard or respected by doctors, dislike of patient-provider communication [58], and anticipating fear/embarrassment [59] prevent people from seeking further care. In previous work, patients viewed negative emotions associated with appointments as having more cost than the benefit of following up [58]. Differently, in the present study, women did place value on care benefits such as resolving vulvar pain, but ultimately said that they would not seek further care because of their past experiences.

While women in this study felt discouraged about seeking further care, differently, doctors reported patients with FGC seeing multiple doctors before finding one they were comfortable with. This is not the first study to report doctor “shopping”, referred to in the biomedical literature as visiting multiple clinicians for the same issue. This is looked upon negatively by doctors, who refer to patients who do this as challenging [60]. This term places blame rather than working to understand what brought the patient to engage in this behavior. Doctor “shopping” in the literature overlooks the emotional component of the clinic visit and the particularities of the everyday lives of those navigating care. It is important to address women’s hesitancy to return to doctors in order to avoid exacerbating existing ailments and undetected complications that may lead to more intensive treatment than would have otherwise been required if treated in a timely manner. Although the current study did not inquire into follow up behaviours, women thought it important to highlight the consequences of their emotional burden by indicating their hesitancy to do so.

Part of what shaped women’s emotional burden was the response to their reproductive bodies. Women and doctors interacted via the doctors’ responses to their bodies, which we suggest may become seen as a boundary object, being acted upon and toward during care [25,26,61]. Just as a road map may lead one reader toward one destination and another reader toward a different one [25], the body may be interpreted as a text that women and doctors read differently.

Women read their bodies in the context of FGC making them “normal” in natal countries but stigmatized in diaspora. This aligns with other literature that describes the tension of no longer fitting in with natal norms and feeling different and othered in host countries [27–29,62]. Differently, OB/GYNs were expertly trained to read the body according to a very broad range of medical “normal” and to bring the body back to this state. These different readings of women’s bodies are situated in a societal context in which FGC is illegal, in which Canadian laws against FGC have fostered ethical dilemmas for OB/GYNs caring for women with FGC [42] and in which FGC is stigmatized and more broadly viewed as something that is not “normal” [39].

The body with FGC has also often been read in the west more generally as “disfigured” [63,64], “mutilated”, not “normal” [39], and, in the current study, a result of what was often perceived as a traumatic experience. This may have contributed to women being referred to mental health care for emotions that might have been perceived as resulting from a trauma. It is important to note that there is a gap in educational resources for addressing sexual and mental health concerns [20]. Therefore, like other professionals who care for women with FGC, mental health care providers likely also lack adequate training and competency for treating this population. The institutional organization of care that prompts the biomedical reading of the body, and the way this differs from women’s reading of their bodies, shaped the emotional healthwork women engaged in to complete appointments.

We found that emotional aspects of care (as they were viewed as relating to stigmatization and mental health) did not fall within the “scope” of obstetric/gynecological work, as evidenced by the referrals to mental health care given to women with FGC when they showed emotional reactions during care. However, these referrals were to no avail since women with FGC who participated in the current research reported that they often did not follow up with mental health referrals. Drawing attention to the ways in which the organization of obstetric/gynecological work shapes emotional healthwork for women with FGC therefore raises the question: How can women’s emotional health be better addressed and cared for? Despite the view that women’s emotional and mental health is taken care of by a multi-disciplinary team, in reality, that team is separated by time and place. We found that women’s emotional health was integrated with the reproductive body, as demonstrated by the emotions that arose in the care context, and flip flopped between reproductive and mental health care, often becoming underacknowledged.

The current model for handling issues of emotion falls under the umbrella term mental health care for which there is no definition which is broadly accepted [65]. Mental health is commonly known as an important component of overall health, whether described as a discipline “to designate a state, a dimension of health,” or as a movement applying “psychiatry to groups, communities and societies” [65 p.115]. Further work is needed to break down this umbrella term, perhaps beginning by better understanding the gradient between emotional health (which OB/GYNs may have the expertise to address) and mental health (for which a mental health specialist may be required).

This is not to suggest that OB/GYNs should be left with the unreasonable task of treating all aspects of a woman’s health. This is also not to suggest that OB/GYNs be trained to treat mental health conditions, nor that yet another task be added to their already loaded plates. However, it is possible that by better distinguishing the multiple facets which produce health inequities, we can begin to better conceptualize and address a broader range of emotions that may be linked with the reproductive body for women with FGC. Although some women may benefit from mental health care, by better conceptualizing a broader range of emotional health as intertwined with the reproductive body, we can work toward more inclusive care for women with FGC in Toronto.

A model for integrated care in New York has been described for survivors of sexual and gender-based violence [66]. This suggests that there is a need for care that integrates reproductive and mental health components. We did not formulate women with FGC under the label of “sexual and gender-based violence survivors”. However, FGC has been discussed using these terms [1]. Further, the elements of reproductive health care that Ades et al. [66] identify as needing better integration with mental health support (i.e., gathering of intake information, performing gynecological examinations) align with the very same aspects of care that women described working hard to navigate (i.e., explaining FGC during intake, getting through vulvar/vaginal examinations). Looking to the reproductive health care experiences of populations beyond women with FGC might therefore teach us something important about many women’s encounters with the system.

Integrated care models such as this one could be used to inspire further integration of emotional health support during reproductive health care for women with FGC. This is important considering that there has been a call across the literature for more culturally informed, holistic care [64,67–69]. However, this proposed model works to address trauma and not all women with FGC may feel that they have undergone a “trauma”. Further, women with FGC may have other related trauma to be considered within their care, including that of migration and “protracted displacement” as well as of stigmatization upon migration to western countries [69–71 p.104]. These considerations of women’s trauma and how they may or may not identify themselves as having underwent various traumas requires further contemplation and perhaps research to best care for women with FGC.

## Conclusion

Overall, this research contributes a better understanding of the ways in which the institutional organization of obstetric/gynecological work practices, beyond any individual doctor, influence the reproductive health care experiences of women with FGC. This is the first study to show the intertwinement of the emotional burden related to the work that women with FGC engage in to navigate reproductive health care and reveals a disjuncture between the reproductive health care needs of women with FGC during appointments (including their emotional needs) and the range of care that OB/GYNs were able to provide. Women’s reproductive health care needs became waylaid by the emotional healthwork in which they had to engage as a result of the organization of obstetric/gynecological work. Women’s emotional reactions (seen by doctors as relating to mental health) were beyond the care that OB/GYNs were able to responsibly provide, which centered around the reproductive body. Therefore, emotional needs intertwined with care often went unaddressed, making medical appointments a difficult experience for women with FGC in the GTA. It is pertinent for future research to investigate this phenomenon in groups without FGC to better understand the ways this extends more generally as something that happens when people traverse reproductive health care. By continuing to better understand the institutional organization of care and how it shapes women’s care experiences, we can work toward more inclusive, comprehensive care that may better address the needs of women with FGC and beyond.

## Supporting information

S1 File Interview guide. (DOCX)
