# Supplementary material for: Reproductive health care appointments: How the institutional organization of obstetric/gynecological work shapes the experiences of women with female genital cutting in Toronto, Canada
Source: PLoS One. 2023 Jan 19;18(1):e0279867. doi: 10.1371/journal.pone.0279867 (PMC9851502; doi:10.1371/journal.pone.0279867)
Supplement: S1 File — (DOCX) [file pone.0279867.s001.docx]

# **Interview Guide/Prompts**

**For Women with FGC**

**Q1:** What do you refer to the practices as (how do you define it)?

*Prompt:* Do you remember when you went through FGM/C?

*Prompt:* How do you feel about it?

**Q2:** Do you have a family doctor? How is your relationship with your doctor?

*Prompt:* How long have you been with your doctor? Why did you stay/leave?

*Prompt:* What do you think about the services you are getting?

**Q3:** Do you go to a specialist for reproductive/sexual health?

*Prompt:* Have you ever been referred to a specialist (OB/GYN)? Why were you referred?

*Prompt:* Was it easy/hard to get a referral when you wanted one?

*Prompt:* Tell me about a time when you went to a reproductive health care specialist.

**Q4:** How was your experience with your doctor?

**This question developed after the first few interviews and in consultation with the CAG**

*Prompt:* When did the doctor find out you had FGC? (Labour? Pap? Check up? Specific issue?)

*Prompt:* What are you looking to get from your provider?

*Prompt:* What are and aren’t you getting from them?

**Q5:** Did your doctor know about FGC?

**This question developed after the first few interviews and in consultation with the CAG**

*Prompt:* What did your doctor say about it? What kinds of questions were they asking?

*Prompt:* How did you deal with how the doctor responded to you?

**Q6:** In general, with your health care, who else is involved in your appointments?

*Prompt:* How do they impact your experience/make your experience better or worse?

*Prompt:* What did they do? What did they say to you?

*Prompt:* Are there any screening measures or other tools used (in-take forms, etc.)?

**Q7:** Do you generally have to pay when you see your doctor or is it covered by OHIP?

**This question developed after the first few interviews and in consultation with the CAG**

*Prompt:* Are you covered by any other programs?

**Q8:** Were you deinfibulated?

*Prompt:* By whom? When did this happen/why?

*Prompt:* Were you given instruction on what would happen and on how to take care of yourself?

*Prompt:* How do you know what deinfibulation is? How did you find out that you could be deinfibulated/that existed as an option for you?

**Q9:** How many children/pregnancies did you have? Tell me about your experience with care related to your pregnancy.

*Prompt:* What kind of clinic were you at? If you were at a high-risk clinic, why?

*Prompt:* Did you follow up with antenatal care? How was that?

**Q10:** What is one reproductive health care experience you could tell me that was good?

**This question developed after the first few interviews and in consultation with the CAG**

*Prompt:* Or any experiences you were not happy with? This is any experience from a-z in the health care system (hospital, clinic, community health centre…)

**Q12:** What would have helped to make your experience better?

*Prompt:* If you had the chance, how would you tell the people in the clinic to improve the situation?

*Prompt:* Were the resources you had helpful/unhelpful?

**Q13:** Have you ever talked about your experience with anyone else? What are the issues that you have heard women talking about in your circle?

**This question developed after the first few interviews and in consultation with the CAG**

*Prompt:* What has your experience been like overall?

*Prompt:* Is there anything else I should know about your experiences in Toronto’s reproductive health care system?

**For Health Care Practitioners**

**Preliminary Questions:**

- How long have you worked in this area? What brought you into doing this work?
- Tell me about your medical school education and residency.
- How often do you see women with FGM/C?
- How do you define the FGM/C types?

**Q1:** What happens when a woman with FGM/C walks into your office? Why might a woman with FGM/C come to see you?

*Prompt:* Take me through step-by-step an encounter with a woman with FGM/C.

*Prompt:* Do you feel prepared to treat her?

*Prompt:* Do you follow any guidelines to treat the woman? How do you know what to do/how to treat her?

**Q2:** When is a specialist required over a general practitioner?

*Prompt:* What is the referral process like?

*Prompt:* How do you feel about referring women with FGM/C? Do you ever hesitate to refer?

*Prompt:* When do you refer? What situations/benchmarks would make you refer a woman with FGM/C to someone else?

**Q3:** What is deinfibulation?

*Prompt:* Can a GP do this? Is this covered by OHIP? Is there only one code for this?

*Prompt:* What are the key steps/decision points when deinfibulating?

**Q4:** Tell me about a time you helped a woman with FGM/C give birth in your practice.

*Prompt:* What is it like when a woman with FGC presents in labour? Do different types impact the way that you work with patients? How is this different from helping a woman without FGM/C?

*Prompt:* How do you decide if you are going to do an episiotomy (or other treatment)?

*Prompt:* Take me through the key steps/decision points.

**Q5**: What is clitoral reconstruction? Is it done here in Canada?

**This question developed after the first few interviews and in consultation with the CAG**

*Prompt:* What boxes would a woman need to check off to be a candidate for reconstruction?

*Prompt:* Take me through step-by-step what would happen from a woman being referred to you to the surgery being complete?

*Prompt:* What contributes to your decision to do the surgery? Any guidelines to follow?

**Q6:** What other guidelines or policies do you have to follow/think about when treating women with FGM/C (hospital/clinic guidelines, government guidelines, association guidelines)?

*Prompt:* Are there any unique forms you have to fill out?

*Prompt:* Anything about the guidelines/policies or forms that you would change? How so?

*Prompt:* Can you show me an example of the policy/protocol?

**Q7**: Do you ever find that there is no OHIP code for a procedure/treatment you have done?

*Prompt:* How would you deal with this/bill OHIP?

**Q8:** Is there something you don’t have in your job, that if you did have, would help you treat women with FGM/C?

*Prompt:* If you were going to build cultural sensitivity into this process, how would you do it?

*Prompt:* What would enhance this?

**Q9:** Have you been asked to perform reinfibulation?

**This question developed after the first few interviews and in consultation with the CAG**

*Prompt:* How do you handle that? What is that process like?

**To Ask Intermittently:**

- Am I getting this right? (Campbell & Gregor, p. 77)
- How do you get that done?
- So, then you did so and so, is that right? (Campbell & Gregor, p. 77)
- What am I missing here? (Campbell & Gregor, p. 77)
- Ask questions at each point in the informant’s narrative “where steps are skipped or discourse words substitute for what actually happens” (Campbell & Gregor, p. 78)
